# Effect of sinusoidal leg cycling exercise period on brachial artery blood flow dynamics in humans

**DOI:** 10.1186/s12576-020-00750-5

**Published:** 2020-04-20

**Authors:** Kohei Miura, Hideaki Kashima, Anna Oue, Ayaka Kondo, Sachiko Watanabe, Masako Y. Endo, Yoshiyuki Fukuba

**Affiliations:** 1 Department of Exercise Science and Physiology, School of Health Sciences, Prefectural University of Hiroshima, Hiroshima, 734-8558 Japan; 2 Department of Health and Nutrition, Faculty of Health Sciences, University of Hiroshima Shudo, Hiroshima, 731-3195 Japan; 3 Faculty of Food and Nutritional Sciences, Toyo University, Gunma, 374-0193 Japan

**Keywords:** Blood flow dynamics, Sinusoidal exercise, Brachial artery, Forearm skin blood flow, Inactive limb

## Abstract

**Purpose:**

To quantify the dynamics of blood flow in brachial artery (BF-BA) in response to sinusoidal work rate (WR) leg cycling exercises of 2-, 4-, and 6-min periods and to examine their relationship with the forearm skin blood flow (SBF).

**Methods:**

Seven healthy young male subjects performed upright leg ergometer exercise with a constant WR (mean sinusoidal WR) for 30 min followed by sinusoidal WR exercise of three different periods (number of repetitions): 2 min (7), 4 min (4), and 6 min (3). The WR fluctuated from 20 W to a peak WR corresponding to 60% peak oxygen uptake (VO_2_). We continuously measured pulmonary gas exchange, heart rate (HR), blood velocity and cross-sectional area of BA, and forearm SBF and sweating rate (SR).

**Results:**

All variables were followed by the sinusoidal WR. The phases of the variables for gas exchange and central circulation, such as VO_2_ and HR with WR forcing were similar (e.g., phase shift (*θ*) in HR [°]: 2 min, 60 ± 7; 4 min, 45 ± 10; 6 min, 37 ± 8; mean ± SD) to previous study results, that is, a longer period showed a shorter *θ* and larger amplitude of responses. Contrarily, the BF-BA response showed anti-phase (approximately 180°) regardless of the period, whereas the *θ* of forearm SBF and SR were similar to gas exchange and central circulation.

**Conclusions:**

Inactive limb BF-BA during sinusoidal leg cycling exercise was out of phase relative to the regulation of O_2_-delivery to active muscles and thermoregulation. The response of BF-BA seems to not always reflect the response of forearm SBF in the downstream area.

## Introduction

Exercise research has primarily focused on blood flow (BF) and its regulatory mechanism(s) of active muscles and/or limbs rather than inactive muscles/limbs. In general, a traditional concept has been widely accepted that BFs to inactive regions, such as inactive muscles and/or limbs, kidneys, and splanchnic organs, are decreased relatively to exaggerate the dramatically increased BFs to active skeletal muscles and the heart with regard to the increment of work rate (WR) (e.g., [1–4]). However, regarding the BF to inactive limbs, the results of recent human studies have been in contrast to this concept, i.e., several studies have found an increase, rather than a decrease in BF, to the inactive limb with the WR of exercise of the contralateral limb [in general, BF in the brachial artery (BF-BA), in the inactive upper limb during leg dynamic exercise] [5–9]. Furthermore, with the continuation of constant WR exercise, a biphasic response in BF-BA has been shown, characterized by an initial decrease followed by a pronounced increase [7, 8, 10]. Specifically, BF-BA decreased transiently during the first few minutes after exercise onset, but subsequently increased beyond the baseline level, and the elevation resulted at least partially from thermoregulatory cutaneous vasodilation (i.e., the elevated skin blood flow [SBF]) following prolonged exercise [8, 11]. On the other hand, initiation of dynamic leg exercise has been found to transiently elicit suppression of BF-BA [8, 10, 12]. Such suppression was mediated by vasoconstriction resulting from increased muscle sympathetic nerve activity [13]. During the continuation of exercise, the initial vasoconstriction was overcome by the cutaneous vasodilative response as a readjustment to thermoregulatory demands, resulting in elevated forearm BF [7].

The BF-BA during the continuation of dynamic leg exercise seems to induce favorable shear stress (SS) response to the endothelium adaptation in inactive vasculatures beyond the active limb (e.g., [6, 14]). The SS in the BA vessel is mainly dependent on the blood velocity (BV) because the shear rate, an estimate of SS, is defined by the BV and diameter (D) and has been shown to be affected mainly by large changes in BV, not small changes in D, during lower limb dynamic exercise [15]. The results from recent studies demonstrating that BF-BA increased following the continuation of dynamic leg exercise suggested a favorable SS profile of the endothelium for the upper limb (e.g., [6, 14]). However, these BF responses were derived from prolonged “constant” WR exercise. Therefore, it remains to be elucidated how BF-BA responds to altered WR during prolonged exercise lasting long enough to reach the approximately steady state of thermoregulatory cutaneous circulation. In other words, from a basic physiology perspective, it should be clarified whether early suppression of BF immediately after the onset of exercise would still appear during the continuation of exercise.

We have frequently used a “sinusoidal” WR forcing function rather than step exercise (i.e., constant WR forcing) to determine cardiorespiratory dynamics in response to exercise [15–19]. Compared with a constant WR forcing function, the sinusoidal WR forcing is continuous, but varies smoothly in WR so that dynamic properties (especially the phase shift) in almost relevant physiological variables can be clearly estimated, and those interrelationships (i.e., faster/slower among the traceability of targeted variables) can also be easily determined [20–22]. In addition, a sinusoidal WR forcing function following prolonged constant exercise (set at mid-WR between low and peak sinusoidal WRs) has an advantage compared with the step-changed constant WR exercise following it because the total work performed during one cyclic sinusoidal exercise remains the same as that of the previously performed constant exercise, yet the WR fluctuates continuously [15]. Accordingly, we used a sinusoidal WR forcing function to determine the BF-BA response to altered WR following 30 min of continued constant exercise, which lasted long enough to reach both the newly increased steady state in BF-BA [7, 8] and a thermoregulatory state [23, 24].

From a basic physiological perspective, we had previously tried to quantify the dynamic property of BF-BA during a leg cycling exercise with prolonged constant WR followed by a 4-min period of sinusoidal WR forcing [15]. The elevated BF-BA during prolonged leg cycle exercise with a constant WR has been postulated to be induced mainly by the increased non-glabrous SBF (i.e., forearm SBF), which is a major downstream circulation (75–80%) for the thermoregulatory adjustment during dynamic leg exercise [11, 25]. Therefore, we hypothesized that during a sinusoidal WR fluctuating leg cycling exercise, the phase delay of BF-BA in response to a WR change would be similar to that of a forearm SBF. Consequently, most variables, including BF variables, adequately followed a sinusoidal form. The forearm SBF showed a phase delay (i.e., ~ 65°) similar to those of the typical cardiorespiratory variables, such as oxygen uptake (VO_2_) and heart rate (HR) (approximately 40°–60°). The phase of BF-BA was, surprisingly, quite different and showed an approximately anti-phasic response (~ 180°), which was apparently dissociated from that in the forearm SBF. Because this phasic dissociation between BF-BA and forearm SBF was only observed during the 4-min period of sinusoidal WR forcing, it is unclear if sinusoidal WR with other periods would induce similar phasic dissociation. Therefore, another study should determine if this anti-phase of BF-BA dynamics would be observed in other period(s) of sinusoidal WR fluctuation. If the BF-BA response always shows an anti-phasic response to change of continuous WR fluctuation regardless of the sinusoidal exercise period, the underlying regulation of BF-BA during leg exercise might be driven by not only thermoregulation, but also by some WR-dependent mechanism(s). The study aim was, therefore, to quantify the dynamics of BF-BA in response to sinusoidal WR leg cycling exercises of 2-, 4-, and 6-min periods and to examine their relationship to the forearm SBF.

## Methods

### Subjects

Nine healthy young male subjects (19–24 years) volunteered for this study. Each subject underwent an initial examination prior to following the main study protocol. During the initial examination, we attempted to measure the subject’s BV by Doppler ultrasonography (detailed procedure described below). However, we were unable to obtain data from two subjects because of the overlapped location between the arterial and venous vessels in the upper-arm target location. Therefore, seven subjects finally participated in the main study. All possible risks associated with participation in the study were explained, and the subjects provided written informed consent. This study was approved by the Ethics Committee of the Prefectural University of Hiroshima and was undertaken in accordance with the Declaration of Helsinki. The subjects had a sedentary lifestyle, performed no regular endurance training, and did not participate in > 4 h of aerobic exercise per week. Their heights and weights ranged from 161 to 172 cm and 53 to 60 kg, respectively.

### Experimental protocol

To test their tolerance limit, the subjects initially performed an incremental ramp exercise test at a rate of 20 W/min on an electromagnetically braked cycle ergometer (232c-XL; Combi Corp., Japan) in a partially recumbent position (approximately 10° behind the vertical upright position) at 60 rpm to estimate ventilatory and gas exchange threshold (VT) and peak VO_2_. VT was estimated by using the V-slope method [26], and gas exchange criteria were used to detect the breakpoints at which there were systematic increases in the ventilatory equivalent for oxygen uptake (VE/VO_2_) and end-tidal partial pressure of oxygen (PETO_2_), with no concomitant increase in the ventilatory equivalent for CO_2_ output (VE/VCO_2_) or decrease in end-tidal partial pressure of CO_2_ (PETCO_2_) [27]. Peak VO_2_ was determined during the last 30 s of ramp exercise. All exercise tests were performed in an air-conditioned laboratory (ambient temperature 22–23 °C, relative humidity 45–55%) situated at sea level. From this preliminary exercise test, the peak VO_2_ and VT of the subjects were 48.1 ± 5.8 mL/min per body weight and 58 ± 2% of peak VO_2_ as mean ± SD.

For the main sinusoidal WR exercise period, each subject rested on the ergometer saddle against a backrest for approximately 30 min prior to exercise. Initial resting measurements were obtained during a 4-min period in which the subject rested in the same position as that used for the ramp exercise; leg cycle ergometer exercise then commenced. An electromagnetically braked ergometer (232c-XL; Combi Corp., Japan) was able to control the WR second by second via a transport cable connected to an external PC software. Both the arms were placed in a relaxed position on side tables that were set approximately at heart level. As a measure of constant WR exercise, the subjects exercised for 30 min at the mean WR of the sinusoidally varying exercise. This was followed by 14-min (sinusoidal WR of a 2-min period times seven repetitions), 16-min (4-min × four), or 18-min (6-min × three) in which WRs were varied between a minimum of 20 W and a peak corresponding to VT (approximately 60% of the subject’s peak VO_2_). The addition of 2- and 6-min periods to our previous study [15] was based on the practical reason to design the protocols. Several specific periods are necessary to perform if the main purpose would identify the transfer function of WR to physiological response [20, 28], this is out of scope of this study. Three different periods of sinusoidal WR exercises were performed in random order at the same time on different days with ≥ 3-day intervals.

### Measurements

Ventilatory and gas exchange parameters (VE, VO_2_, VCO_2_, PETO_2_, and PETCO_2_) were determined breath by breath using a computerized metabolic measuring system (Aero-Monitor; Minato Medical Science, Japan). Prior to each exercise test, the flow sensor and gas analyzers were calibrated by introducing a known volume of air at several mean flow rates and gas mixtures of known composition, respectively. The second-by-second time course was calculated for each variable by interpolation of the breath-by-breath data.

SBF was monitored in the center of the right forearm by using a laser-Doppler flowmeter (ALF21; Advance Co., Ltd., Japan) as a measure of red blood cell flux. The forearm skin SR was determined in an enclosed location by capacitance hygrometry, calculated from the relative humidity and temperature (THP-B3T; Shinei, Japan) of the air flowing out of a 12.56-cm^2^ capsule at a rate of 1.5 L/min [29]. The reproducibility of the SBF measurement using the laser-Doppler flowmeter was confirmed in the previous studies (coefficient of variance: ~ 20%) [30–32]. The variables described above were converted into digital data by using an analog-to-digital (AD) conversion device and software (PowerLab 8/35, ADInstruments, Colorado Springs, CO, USA) at 1 kHz. Then, second-by-second time courses were calculated for each variable by interpolating the beat-by-beat or average data.

Beat-by-beat BV through the right BA to the distal third of the right inactive upper limb, as well as vessel diameter, was measured by using a pulse-echo Doppler ultrasound (LOGIQ S6; GE Medical Systems, Japan) and a linear 5.0-MHz probe with an insonation angle of < 60°. The diameter of each vessel was measured simultaneously with an imaging frequency of 12.0 MHz. The sample volume was positioned in the center of the vessel and adjusted to cover the full BA diameter. For every cardiac cycle, the Doppler tracing was analyzed by using integral software to obtain the antegrade and retrograde velocities (mean velocity = antegrade − retrograde velocity) in the BA. The BF-BA was calculated from the BV and cross-sectional area of the vessel, as previously described (e.g., [33–35]). Briefly, audio-range signals for the antegrade and retrograde velocities reflected from the moving blood cells, as well as the electrocardiogram (ECG) signal, were digitally sampled by using a 20-kHz AD conversion (PowerLab 8/30, ADInstruments, Colorado Springs, CO, USA). Audio-range signal spectra were processed offline by Doppler signal processing software [using a fast Fourier transfer analysis and a 256-point Hamming window (12.8 ms each)] to yield instantaneous antegrade and retrograde velocities. Velocity signals were recorded at 100 Hz on a computer system, in addition to the ECG, so that beat-by-beat data could be analyzed. Finally, second-by-second time courses of antegrade, retrograde, and mean (i.e., net) velocities were calculated by interpolation of the beat-by-beat data. B-mode echo images of the right BA were recorded simultaneously on a hard-disk drive video recorder, and the diameter of the vessel was measured by using on-screen calipers. Vessel diameters were summarized at rest every 5 min during the first 30 min of constant WR exercise and at 10-s intervals during the sinusoidal exercise. The validity of the simultaneous pulsed and echo Doppler ultrasound system for the BF measurement in this study was already conducted previously including the confirmation using a phantom artificial flow [35]. In this study, the mean BF (i.e., net BF) was the main variable discussed as BF-BA because the retrograde BF was not able to fit the sinusoid in some cases because of the low S/N ratio, as described in a previous study [15].

### Model fitting

To determine the dynamic characteristics of each variable relative to sinusoidal exercise, the amplitudes of fluctuations and phase shifts were calculated. The sec-by-sec variables were superimposed every period (excepting the first one: second–seventh, second–fourth, and second–third in 2-, 4-, and 6-min periods, respectively) to fit the sinusoidal model as follows. The first period was excluded because the response during this period theoretically includes a transient non-sinusoidal component. A sinusoidal model was used to broadly describe the response,* y*(t):

1 $$y\left( t \right) = M + A \times {\text{sin}}\left[ {\left( {\frac{2\pi }{T}} \right) \times t - \theta } \right],$$

where *t* = time, *T* = period (of sinusoidal WR; i.e., 120, 240, or 360 s for 2-, 4-, or 6-min periods, respectively), *M* = mean level, *A* = amplitude, and *θ* = phase shift. Curve fitting was performed by using the least-squares technique (SigmaPlot ver. 12, Systat Software Inc., San Jose, CA, USA). The square of the correlation coefficient (r) was used as a conventional index of goodness of fit. The ratio of A to M [A/M (%); × 100] was defined as the relative amplitude of the response. The sinusoidal model fit was considered to be successful for *r*^2^ > 0.5.

### Data analysis

Values are expressed as the mean ± SD. The time course of changes in each variable was assessed by using a two-way repeated-measures analysis of variance (ANOVA) with two factors of time and period. The analyses showed significant time effects of the principal variables without any period effect so that subsequently, the time ranges were divided into two categories for the one-way ANOVA with a factor of time. First, the change during the first 30 min of constant exercise (the time bins were 4–6 and 28–30 min) with rest was tested. Second, the change throughout the sinusoidal exercise (the time bins were roughly the middle and last 2-min: 6–8, 7–9, and 8–10 min and 12–14, 13–15, and 14–16 min in 2-min, 4-min, and 6-min periods, respectively) against the values at the end of constant exercise (i.e., at 28–30 min) was separately tested. When a significant difference was detected, it was further evaluated by using Tukey’s post hoc test. Thereafter, the difference(s) in periods (i.e., 2-, 4-, and 6-min periods of sinusoidal WR conditions) among the estimated parameters (θ or A/M) within each principal variable were tested by using one-way ANOVA with a factor of period, coupled with Tukey’s post hoc test. Statistical significance was accepted for P < 0.05. All statistical procedures were performed by using SPSS version 18.0 for Windows (SPSS Inc., Armonk, NY, USA).

## Results

At the end of the first 30 min of constant WR exercise, almost all variables attained steady-state responses, including not only the gas exchange variables, but also the SBF and SR, with no further increases during sinusoidal exercise (Table 1). The HR showed a rapid increase during 30 min of constant exercise and a further small increase during the sinusoidal exercise (Table 1). During the first 5 min of constant WR exercise, the BF and diameter of the BA were reduced, which then recovered and increased beyond the baseline level at the end of the first 30 min, and no further increases were observed (Table 1).

**Table 1:** Ventilatory, pulmonary gas exchange, and circulatory measurements at baseline and during constant and sinusoidal work rate (WR) exercise periods

Period	Baseline	Constant WR exercise		Sinusoidal WR exercise	
		4–6 min	28–30 min	Middle 2-min*	Last 2-min*
VE, L/min					
2-min	9.8 ± 1.9	25.4 ± 3.7a	27.0 ± 3.9a	26.9 ± 5.2	27.2 ± 3.6
4-min	9.6 ± 1.7	25.0 ± 3.2a	27.3 ± 3.4a,b	27.8 ± 2.8	27.7 ± 2.9
6-min	9.5 ± 1.5	25.8 ± 4.0a	26.4 ± 4.1a	27.0 ± 3.5	27.4 ± 3.1
VO_2_, mL/min					
2-min	223 ± 18	913 ± 62a	933 ± 87a	940 ± 82	935 ± 78
4-min	221 ± 29	942 ± 70a	968 ± 79a	967 ± 71	949 ± 62
6-min	189 ± 34	928 ± 78a	940 ± 101a	948 ± 96	938 ± 99
VCO_2_, mL/min					
2-min	199 ± 30	793 ± 71a	842 ± 90a,b	877 ± 74	866 ± 80
4-min	192 ± 22	811 ± 65a	892 ± 78a,b	886 ± 70	880 ± 71
6-min	202 ± 38	809 ± 77a	869 ± 88a,b	867 ± 92	871 ± 101
HR, bpm					
2-min	71.9 ± 9.8	105.3 ± 10.2a	110.2 ± 11.7a,b	114.9 ± 10.8c	116.2 ± 11.3d
4-min	70.3 ± 12.7	106.8 ± 11.0a	109.0 ± 11.3a	113.2 ± 12.3	115.0 ± 13.8d
6-min	73.1 ± 10.5	106.0 ± 9.9a	110.8 ± 12.5a	114.0 ± 13.1c	117.0 ± 12.5d
BF-BA, mL/min					
2-min	78.5 ± 28.2	50.7 ± 24.8a	102.7 ± 29.8a,b	105.5 ± 29.4	102.3 ± 29.6
4-min	77.9 ± 33.4	52.2 ± 26.3a	98.9 ± 28.4a,b	102.2 ± 37.1	100.8 ± 35.9
6-min	70.3 ± 25.8	49.9 ± 27.7a	100.2 ± 32.0a,b	101.8 ± 32.0	105.0 ± 30.4
BA-diameter, cm					
2-min	0.369 ± 0.022	0.350 ± 0.038a	0.382 ± 0.040a,b	0.384 ± 0.029	0.383 ± 0.031
4-min	0.373 ± 0.029	0.349 ± 0.040a	0.379 ± 0.038a,b	0.381 ± 0.033	0.381 ± 0.028
6-min	0.370 ± 0.021	0.345 ± 0.029a	0.380 ± 0.029a,b	0.381 ± 0.024	0.382 ± 0.030
SBF-forearm, a.u.					
2-min	2.2 ± 1.1	4.0 ± 1.9	9.8 ± 4.2a,b	10.9 ± 4.1	11.6 ± 3.9
4-min	2.7 ± 1.4	3.9 ± 2.5	9.9 ± 3.0a,b	10.5 ± 3.8	11.4 ± 3.2
6-min	2.0 ± 1.0	4.2 ± 1.9	11.2 ± 3.5a,b	11.1 ± 3.7	12.3 ± 4.4
SR-forearm, mg/min					
2-min	0.07 ± 0.02	0.10 ± 0.04	0.29 ± 0.14a,b	0.31 ± 0.10	0.30 ± 0.22
4-min	0.05 ± 0.03	0.09 ± 0.03	0.28 ± 0.12a,b	0.28 ± 0.19	0.27 ± 0.22
6-min	0.05 ± 0.02	0.08 ± 0.03	0.30 ± 0.11a,b	0.30 ± 0.22	0.29 ± 0.19

Values are mean ± SD

* Middle and last 2-min: 6–8, 7–9, and 8–10 min and 12–14, 13–15, and 14–16 min in 2-min, 4-min, and 6-min periods of sinusoidal WR exercises, respectively

a: baseline vs. 4–6 min and baseline vs. 28–30 min, b: 4–6 min vs. 28–30 min, c: 28–30 min vs. middle, d: 28–30 min vs. last 2-min (*P* < 0.05)

**Fig. 1:**
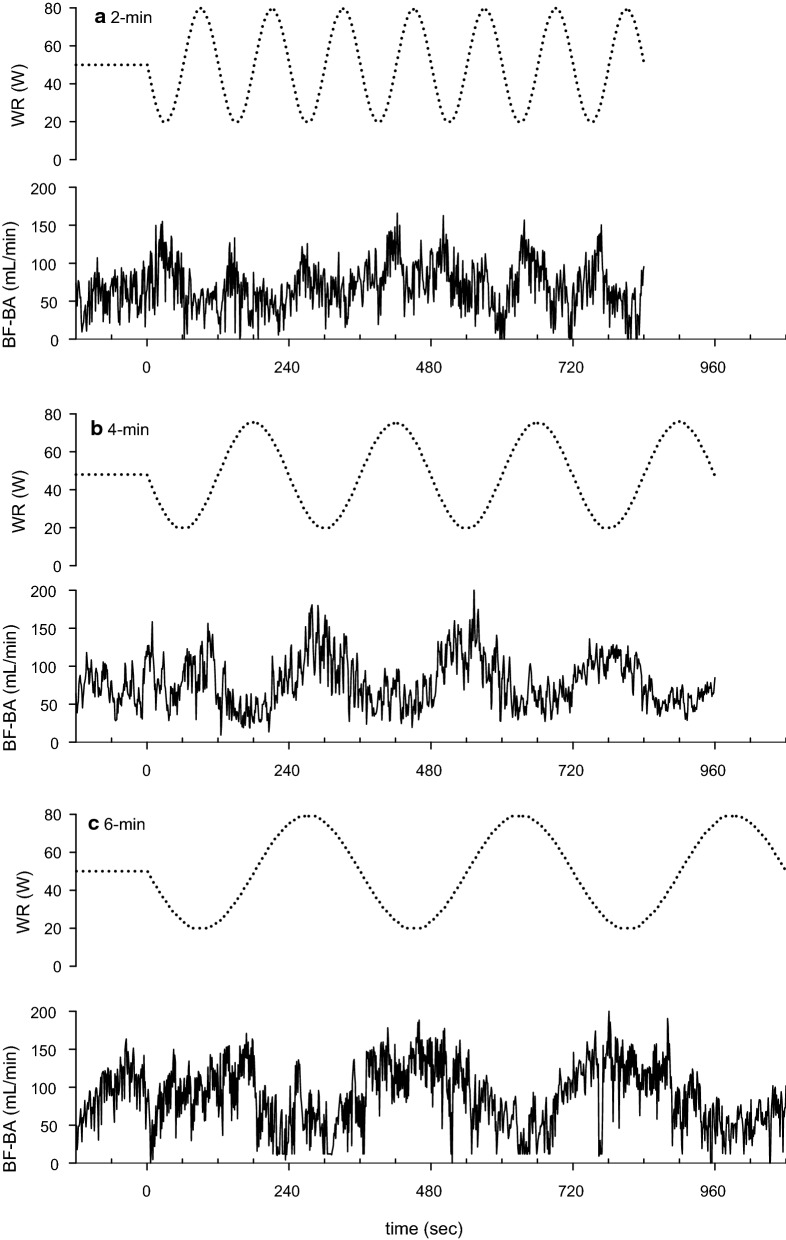
Example of the time-serial changes of BF-BA during sinusoidally fluctuating WR exercise with the periods of **a** 2 min, **b** 4 min, and **c** 6 min in a representative subject. Time “0” indicates the onset of sine exercise. The dotted line indicates the sinusoidal WR change as a reference

**Fig. 2:**
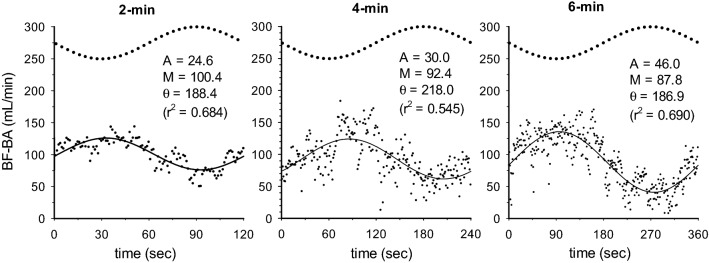
Example of the superimposed responses of BF-BA (blood flow in brachial artery; sec-by-sec data expressed as each dot point) in 2-, 4-, and 6-min periods of sinusoidal WR forcing (bold dotted line as a reference) in the representative subject. The results by fitting of sinusoidal curve are shown by the thin line (see details in “Model fitting” section)

**Fig. 3:**
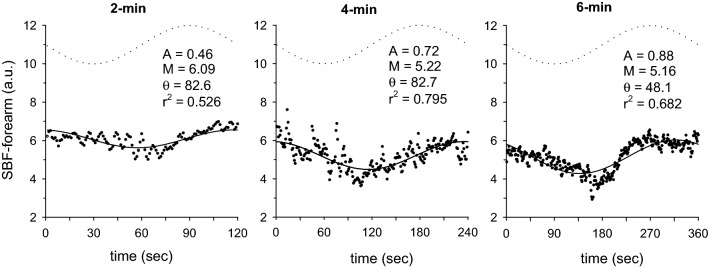
Example of the superimposed responses of SBF-forearm (skin blood flow in the non-glabrous forearm; sec-by-sec data expressed as each dot point) in 2-, 4- and 6-min periods of sinusoidal WR forcing (bold dotted line as a reference) in a representative subject. The results by fitting of sinusoidal curve are shown by the thin line (see details in “Model fitting” section)

In Fig. 1, the BF-BA shows a sinusoidal pattern during the sinusoidal WR exercise in each of the 2-, 4-, and 6-min periods. Therefore, a sinusoidal model with a fixed period was fitted to each response. Examples of the model fits for BF-BA during 2-, 4-, and 6-min sinusoidal WR exercises are shown in Fig. 2. In this instance, Eq. 1 was fitted to the BF-BA which was averaged by superimposing the sec-by-sec data obtained during the second to seventh, second to fourth, and second to third sinusoidal exercises with 2-, 4-, and 6-min periods. The BF-BA responses were nearly sinusoidal (mean *r*^2^ = 0.684, 0.545, and 0.690 during the 2-, 4-, and 6-min periods, respectively). The fluctuation in BF-BA appeared as a mirror-image response to the sinusoidally varying WR (Figs. 1 and 2). The forearm SBF responses also showed a sinusoidal pattern (mean *r*^2^ = 0.526–0.795), but those were followed by sinusoidal WR fluctuation with tighter delays (e.g., Fig. 3).

**Fig. 4:**
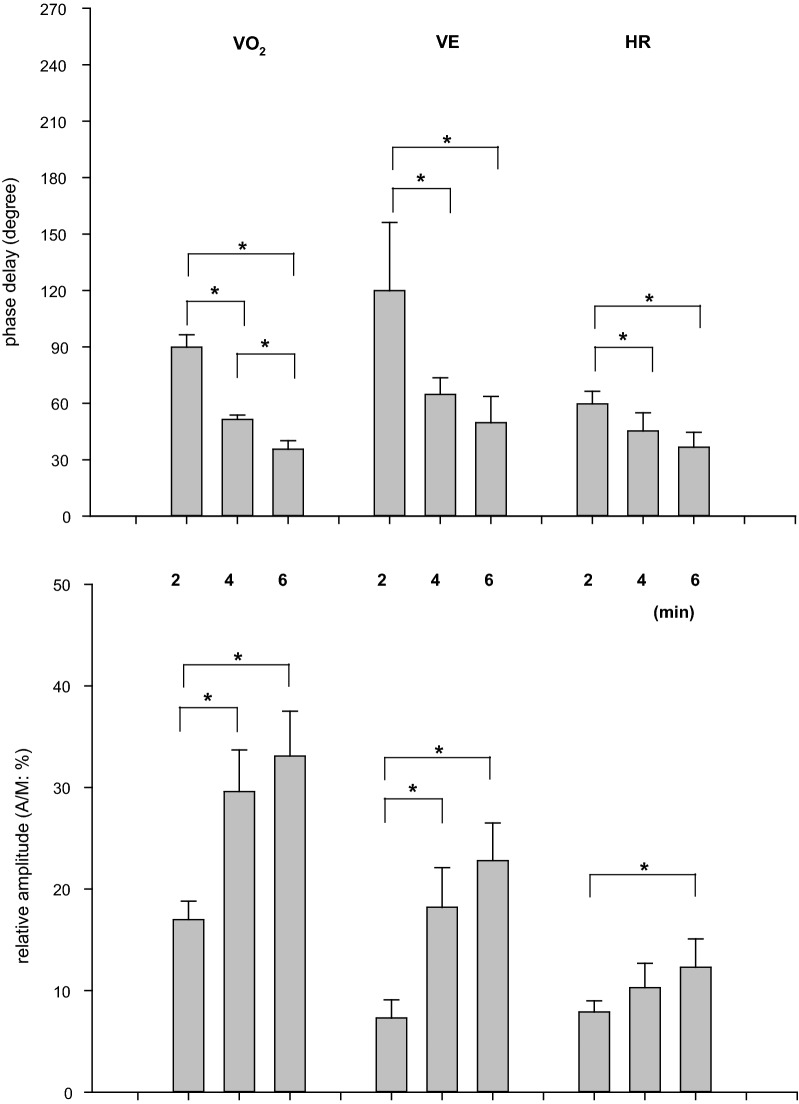
The effect of the periods of sinusoidal WR on the phase delays and relative amplitudes (A/M) of typical gas exchange and circulatory variables: VO_2_, VE, and HR. Data are expressed as the mean ± SD. Significant differences between the periods are indicated by * for *P* < 0.05

As expected from the previous sinusoidal WR studies [15–18, 28, 36], the ventilatory, gas exchange, and HR responses closely followed the sinusoidal WR pattern regardless of period, accompanied by the expected phase delays. The phases and A/Ms for VO_2_, VE, and HR; i.e., the fundamental O_2_-transport-related variables, were similar to those in previous studies with sinusoidal WR forcing (Fig. 4). For example, the phases of VO_2_ in the 2-, 4-, and 6-min periods were 90 ± 7, 52 ± 3, and 35 ± 4 (°), and the A/Ms were 17.0 ± 1.8, 29.6 ± 4.1, and 33.1 ± 4.4 (%), respectively. The circulatory principal variable, HR, also showed similar characteristic results (phases: 60 ± 7, 45 ± 10, and 37 ± 8 (°); A/M: 8.0 ± 1.1, 10.3 ± 2.4, 12.3 ± 2.8 (%) in the 2-, 4-, and 6-min periods, respectively).

**Fig. 5:**
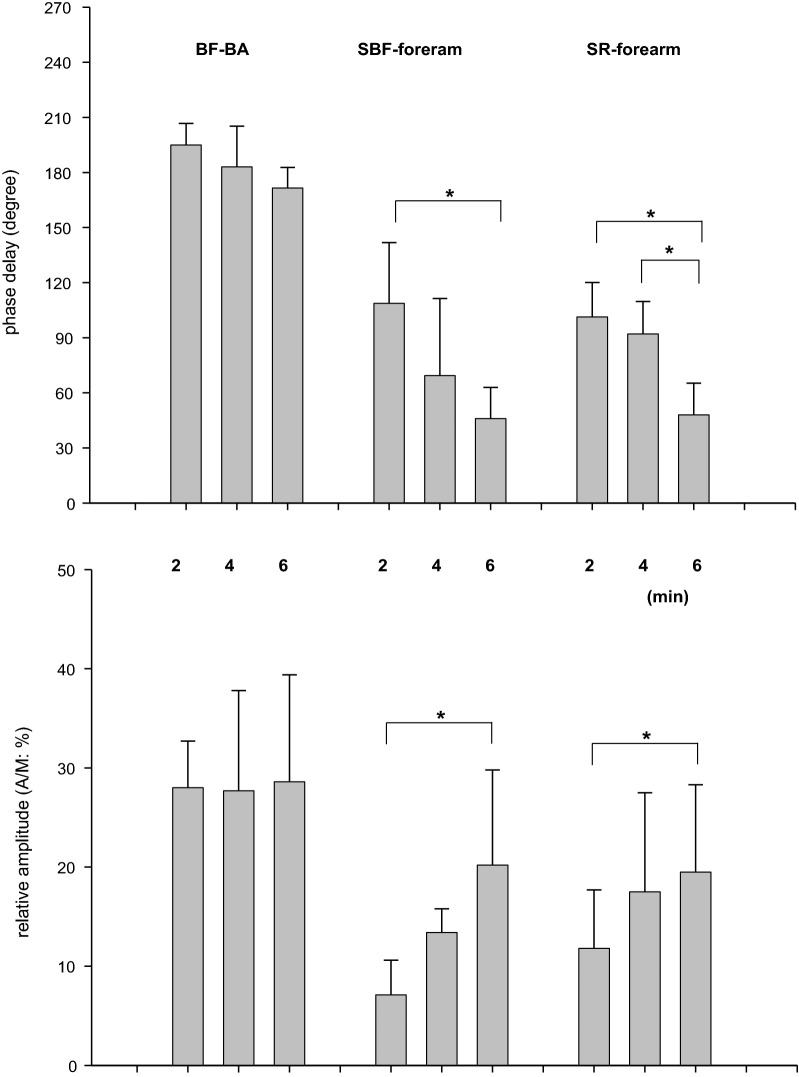
The effect of the periods of sinusoidal WR on the phase delays and relative amplitudes (A/M) of variables in upper non-active limbs: BF-BA, SBF, and SR in the forearm. Data are expressed as the mean ± SD. Significant differences between the periods are indicated by * for *P* < 0.05

With respect to the variables in the non-active limb (upper arm), the phases and A/Ms for SBF and SR in the forearm also showed results similar to those in the fundamental O_2_-transport related variables (Fig. 4). That is, a longer period of sinusoidal WR forcing showed a shorter phase and larger amplitude of both responses (Fig. 5). For example, the phases of SBF in the forearm in 2-, 4-, and 6-min periods were 108 ± 33, 69 ± 42, and 46 ± 17 (°), and the A/Ms were 7.1 ± 3.5, 13.4 ± 2.4, and 20.2 ± 9.6 (%), respectively. In contrast, the response of BF-BA showed an approximately anti-phase (approximately 180°) and a relatively constant A/M (~ 28%) regardless of the period of sinusoidal WR forcing. The phases of BF in the BA in the 2-, 4-, and 6-min periods were 195 ± 12, 183 ± 22, and 172 ± 11 (°), and the A/Ms were 28.0 ± 4.7, 27.7 ± 10.1, and 28.6 ± 10.8 (%), respectively. These characteristic results in the BF-BA were apparently inconsistent with those in the downstream main BF (i.e., forearm SBF).

## Discussion

In this study, there were two key findings. First, regardless of the period (2-, 4-, and 6-min) of sinusoidal exercise in the moderate exercise domain, the responses of BF-BA consistently showed an approximately anti-phase (i.e., ~ 180°) and a relatively constant amplitude. Second, the responses in the forearm SBF, a major area downstream of the BF-BA, were similar to phases and amplitudes of the gas exchange and central circulation (that is, a longer period showed a shorter phase and larger amplitude), and were not compatible with the responses of the BF-BA. These results suggested that the dynamics of the BF-BA seems to be unrelated to the forearm SBF during sinusoidal leg cycling exercise.

Sinusoidal WR forcing exercise with different periods is not a new approach to the study of ventilatory, gas exchange, and HR dynamics during exercise in the sub-VT domain [16–18, 28, 36] or even in the supra-VT domain [19]. For example, during 4 min of sinusoidal WR fluctuation in the sub-VT exercise domain using a protocol and exercise domain similar to those used in the present study, the phase delays were 56°–83°, 42°–60°, and 24°–66° in VE, VO_2_, and HR, respectively [28]. As shown in Fig. 4, the results in the present study were consistent with those reported in previous studies. The usage of the different sinusoidal periods showed that a longer period was associated with a shorter phase and larger amplitude of response if the underlying control system operates in a similar manner, which is consistent with the well-known exponential dynamics in response to step WR exercise [20, 22]. In the present study, experiments using 2-, 4-, and 6-min periods were conducted and showed expected results roughly consistent with the results (e.g., 2 min, 80°–107°; 4 min, 42°–60°; and 6 min, 24°–46° in VO_2_) of a previous study [28]. Similar to the phasic and amplitude responses of VO_2_ and HR, the forearm SBF and SR showed smaller phases and larger amplitudes when the period was longer (Fig. 5). Surprisingly, the response of BF-BA was apparently different from other basic variables of O_2_ transport, such as VO_2_ and HR. That is, the response of BF-BA was approximately anti-phase and had a relatively constant A/M regardless of the period of sinusoidally altered WR within 2- to 6-min periods. This is a major novel finding of the present study.

A cardiovascular adjustment to the thermoregulatory demands of sustained constant WR exercise [37, 38] leads to cutaneous vasodilation in the non-glabrous region of the limb (see the later explanation) when leg cycling exercise is prolonged and is proportionally related to the increase in core temperature [25, 39]. In general, 75–80% of BF-BA is directed to the skin circulation for the thermoregulation [11, 25], whereas only approximately 30% is directed to the skin at rest under thermoneutral condition [40]. Recently, Simmons et al. [7] revealed that the BF-BA during the long-lasting constant WR cycling exercise (~ 30 min) was affected by the downstream non-glabrous SBF using an excellent maneuver involving the local cooling of the forearm and hand that was adopted after the 30 min for exercise onset. In the non-glabrous skin such as the forearm and dorsal hand, the reflex control of the skin vasculature is mediated by two sympathetic pathways: a noradrenergic vasoconstrictor system and an active vasodilator system, whereas in the glabrous (non-hairy) such as the palm, the reflex control is mediated by a noradrenergic vasoconstrictor system only [41]. Such a fundamental finding naturally postulates that the BF-BA dynamics would be determined mainly by the downstream BF to the non-glabrous skin region; i.e., the forearm SBF. The present result, however, failed to prove this hypothesis under non-constant continuously changing conditions of WR (i.e., sinusoidal forcing). Therefore, the present results indicate that, while the achieved steady-state level of the BF-BA itself with a continuation of constant WR exercise seems certainly to be determined by the BF to non-glabrous skin by the thermoregulatory adjustment, other downstream BF(s) affect the BF-BA, at least, if the WR is continuously changing.

The forearm SR also responded in a manner similar to the dynamics of the forearm SBF (Fig. 5). Previously, Yamazaki et al. [42] showed that the forearm SR followed well the sinusoidal WR changes of three different periods and the amplitudes were bigger and the phase lags were shorter as the WR periods were longer. In addition, those researchers continuously measured the esophageal temperatures (*T*_es_) and speculated that the larger amplitude of *T*_es_ with a longer period of sinusoidal WR forcing affected the SR as a thermal factor of body temperature regulation with some contamination of non-thermal factor(s). We could not measure the core temperature; however, the forearm SR dynamics were similar to those of SBF because both the SBF and SR of the forearm seemed to be mainly induced by the central regulation of the dynamics of core temperature. The present results mean that similar forearm SBF and SR responses are responsible for supplying more blood to the sweat glands and skin surface in non-glabrous areas because the non-glabrous SR was strongly dependent on the core temperature [43], and the increase in non-glabrous SBF with increased *T*_es_ was apparently larger than that in the glabrous skin [44].

The response of the BF-BA is determined by both the central mean arterial blood pressure (MAP) and peripheral vascular conductance (VC). Although the present study did not measure those directly because of the usage restriction of the noninvasive beat-to-beat MAP measuring device, our previous study had already explored and clarified that the VC of the BA showed a similar anti-phasic shift (~ 175°) and a consistently large amplitude, whereas the phase of MAP was dissimilar (~ 30°) and was instead similar to the changes in HR [15]. Therefore, the anti-phasic response of the BF-BA during 4-min sinusoidal WR exercise appeared to be principally peripheral in origin. In other words, the changes in BF-BA during one cycle of sinusoidally changed WR exercise were mainly associated with concomitant vasodilation/vasoconstriction of the resistance vessels within the forearm and hand.

The BA supplies most blood to the skin and skeletal musculature in the forearm and hand. Although a huge area of skin in the upper limb distal to the elbow is non-glabrous in the forearm and dorsal hand where the thermoregulation is substantially exerted, the phases in the non-glabrous (i.e., forearm) SBF were dissociated with those in the BF-BA (Fig. 5). The dynamic characteristics of the SBF and SR in the skin of the inactive upper limb during sinusoidal leg exercise were previously examined to establish the effect of exercise on thermoregulation [23, 24, 42]. Yamazaki et al. [42] observed the responses of the body, skin temperatures, and forearm SR and showed that the SR phase (~ 63°) during 4-min periods of leg cycling always preceded those of the sinusoidally slightly varying body and skin temperatures. Their subsequent study explored the responses in reflex control between the glabrous and non-glabrous skin of the upper limb during leg sinusoidal cycling [23]. The SBF responses in the non-glabrous (i.e., forearm) skin showed a sinusoidal pattern and followed the cyclic changes in WR with a similar phase delay (~ 70°), whereas the response of glabrous skin (i.e., palm) was not clearly sinusoidal due to abrupt large fluctuations and sudden irregular dip-like reductions. The responses in the forearm SR and SBF during the 4-min period in the present study were consistent with their results. The present results indicated that the dynamics of SBF in the forearm did not explain those of the BF-BA. The question remains, however, whether SBF in a glabrous area (i.e., the palm) may have a specific role in the dynamics of the BF-BA because the non-glabrous and glabrous SBF responses in the forearm and hand were at least different during exercise [23]. Although we tried to measure the SBF in the palm in a preliminary experiment, the responses were very noisy, especially because of the sudden dip-like reduction with reflex vasoconstrictions presumably due to non-thermal factors, and could not adequately fit the sinusoidal curve for the small number of repetitions. This is one of the limitations in the present study; so, to accurately identify the relationship of palm SBF to the upstream BF-BA remains to be fully elucidated by experiments with a greater number of repetitions for sinusoidal WR.

Another potential candidate is the inactive skeletal musculature in the forearm and hand. It is technically difficult to measure the BF to the skeletal musculature separately from the BF to the skin except by using more invasive methods, such as measuring [^125^I] antipyrine clearance. Using this technique, Johnson and Rowell [25] estimated the relative contributions of skin BF and inactive muscle BF during prolonged constant WR leg cycling and showed that skin and muscle BFs gradually increased and decreased with time, respectively [25]. In addition, Ooue et al. [11] showed that indirect estimation by the simultaneous measurement of venous outflows originating mainly from the skin (surface) and musculature (deep) regions indicated that the arterial inflow (i.e., BF-BA) to the upper limb during leg cycling exercise might be diverted to some extent from the muscle to the skin. Thus, both studies suggest that a larger proportion of the arterial inflow in the BA to the inactive limb is directed to the skin. This flow seems to be dependent on the thermal environment. However, there is a possibility that the BF to non-exercising muscular beds in the forearm and hand may induce an anti-phasic BF-BA response. The relatively constant A/Ms of anti-phasic BF-BA dynamics during three different periods postulate to be derived from frequency-independent manner (i.e., constant response regardless of the period) as well as from more rapid neural-driven factors. The magnitude of MSNA is expected to be simultaneously followed with WR change and may exert to induce a WR-dependent change in rapid vasoconstriction and vasodilation that affects the relative changes in muscle BF and affects the upstream BF-BA. This issue remains to be resolved.

Evidence is accumulating that aerobic exercise training mainly using the lower limbs, such as cycling and walking, induces a favorable vascular adaptation in inactive upper limbs [6, 14, 45]. However, the vascular mechanisms whereby such nonspecific or systemic effects occur in the inactive upper limbs of individuals undergoing leg exercise training have not been fully elucidated [6]. Acute exercise-derived vascular adaptation is essential to obtain the benefit(s) from exercise training. Presently, the most plausible candidate mechanism is that the exercise induced elevation of SS, i.e., the response is approximately consistent with the BF profile in the upper limb, may have substantial effect(s) via the endothelial adaptations [46]. Therefore, the present study was warranted from a basic physiology perspective. In other words, these results contribute to the literature by providing insight into the mechanism(s) underlying an acute vascular adaptation by the detailed BF dynamic properties in the inactive upper limb during an actual training exercise that mainly uses the lower limbs.

## Conclusion

Thus, the responses of forearm SBF and SR are similar to the response of gas exchange and central circulation to sinusoidal WR fluctuation, with a longer period being associated with a shorter phase and larger amplitude of responses. On the other hand, the response of BF-BA was anti-phase and had constant amplitude regardless of the period. These results suggest that the dynamics of BF-BA seems not to be always reflected by the forearm SBF, but by the BF in the palm and/or inactive skeletal muscles in response to WR change after exercising in a sufficiently long duration (i.e., to attain the approximately steady state of thermoregulatory readjustment).

## Data Availability

Not applicable.

## Abbreviations

ANOVA: Analysis of variance
BF: Blood flow
BF-BA: Blood flow in brachial artery
BV: Blood velocity
D: Diameter
ECG: Electrocardiogram
HR: Heart rate
MAP: Mean arterial blood pressure
MSNA: Muscle sympathetic nerve activity
PETO_2_: End-tidal partial pressure of oxygen
PETCO_2_: End-tidal partial pressure of carbon dioxide
SBF: Skin blood flow
SD: Standard deviation
SR: Sweating rate
SS: Shear stress
*T*_es_: Esophageal temperatures
VC: Vascular conductance
VE/VCO_2_: Ventilatory equivalent for carbon dioxide output
VE/VO_2_: Ventilatory equivalent for oxygen uptake
VO_2_: Oxygen uptake
VT: Ventilatory and gas exchange threshold
WR: Work rate
